# Mr. Jenkinson on the Use of Myrrh, Steel, &c.

**Published:** 1802-07

**Authors:** 


### *To the Editors of the Medical and Phyfical Journal.*

Gentlemen,

Dr WILLAN, in common with moft other practitioners in this country, appears to be fully acquainted with the good effects of the medicine known by the name of Griffiths's Mixture; and has devifed an imitative preparation of iron, (ferrum præcipitatum) to which he attributes peculiar efficacy. This preparation, which my flight knowledge of chemiftry will not furnifh me with one reafon for fuppofing materially different from the ferri rubigo of the fhops, may be exhibited in a pilular form, and the naufeous liquid one of the original medicine medicine thus avoided. The advantages of ſuch a change are, no doubt, of great conſequence in practice, for ſeveral reaſons not neceſſary to be detailed here, as well with regard to other nauſeous medicines as this. We need not, however, content ourſelves with getting one ingredient into this manageable ſtate; for nothing can be more eaſy than to attach both the myrrh and the kali to vitriolated iron *in a pilular form*, preſerving exactly the proportions of Dr. Griffiths, and of courſe every thing which can poſſibly aſſiſt in producing the effects of the mixture. This I have for ſome time been in the habit of doing, without loſing any of the efficacy of the old compound; and as I have never heard or read of it being done elſewhere, I have deemed the matter worthy of being communicated to you; how juſtly, your inſertion or rejection of my letter will determine. My firſt formula, the principle of which needs no explanation to thoſe who have read what Dr. Percival, and after him Mr. Chamberlain, have told us reſpecting the ſolvent property of camphor, is the following:

℞. G. myrrh, pulv. ʒj. Kali præpar. ʒſs. Ferri vitriolati, gr. xv. G. camphoræ, (ſp. v. ſub.) gr. viij. hæc contunde in maſſam, et divide in pilulas xviij. quarum tres quatuorve ſimul adultis ſumendæ ſunt.

Rather more than uſual pains muſt be beſtowed, as the maſs ſometimes requires to be worked between the fingers before it will aſſume a proper conſiſtence. All aqueous moiſture being thus avoided, the alkali, partly enveloped in the body of the maſs, and partly defended from the air by the flour the pills are rolled in, has never, to my knowledge, ſhewn any tendency to liquefaction; if it ever ſhould, moiſtening the fingers when rolling the pills, with mucilage of gum arabic, which dries almoſt inſtantaneouſly, and leaves a coat of gum arabic over the whole ſurface it is applied to, will prevent it effectually. [This mode of coating pills I have often uſed when any of their ingredients have been of a volatile nature, with great advantage; even ammonia præparata, thus defended, may be employed in a pilular form, and will keep very well, as I have repeatedly experienced with much ſatisfaction.] Eighteen pills may appear too ſmall a number for the weight of the maſs; but, if properly made, it need not be divided into more on account of ſize, which, at this number, or even ſixteen, is very moderate. I have another mode of making theſe articles into pills, equally convenient and eaſy, where camphor is inadmiſſible; and this conſiſts ſimply in ſubſtituting a few drops of *pure* alkohol for the camphor ſet down in the preceding formula. I need hardly obſerve, that opium, pulvis ſcillæ, or any other drug, uſed in ſmall quantities, may be added at plea-

ſure, without detriment to the maſs. Before I conclude, I cannot avoid relating a fact, which leads me to ſuppoſe, that the remedy, which is the ſubject of this letter, has not yet been employed in all the diſeaſes it is capable of relieving. A country patient of mine, who had taken it in a pilular form for a threatening pulmonary affection with complete ſucceſs, recommended its uſe to a neighbour, long afflicted, as I afterwards found, with a dyſpepſia which had baffled the efforts of more than one eminent practitioner, and with the ſame reſult as in her own caſe. Convinced, as you muſt be, that the ſlighteſt improvements in pharmacy are of importance to the intereſts of medicine, (for what avails the ſkill of the greateſt phyſician when the ſuitable remedies are too nauſeous to be properly perſevered in?) you will not condemn me for my minuteneſs if I have never totally loſt ſight of utility.

*Mancheſter, June* 2, 1802.

